# Serum STARD4-AS1 as a Novel Marker for Gastric Cancer Diagnosis and Promotes Gastric Cancer Progression

**DOI:** 10.14309/ctg.0000000000000915

**Published:** 2025-09-03

**Authors:** Xiuyu Chu, Min Cao, Xinyue Qin, Xian Li, Ming Zheng, Xianjuan Shen, Shaoqing Ju

**Affiliations:** 1 Department of Laboratory Medicine, Affiliated Hospital of Nantong University, Medical School of Nantong University, Nantong, China;; 2 Research Center of Clinical Medicine, Affiliated Hospital of Nantong University, Nantong, Jiangsu, China;; 3 Department of Laboratory Medicine, Gusu School, The Affiliated Suzhou Hospital of Nanjing Medical University, Suzhou Municipal Hospital, Nanjing Medical University, Suzhou, China;; 4 Department of Laboratory Medicine, Northern Jiangsu People's Hospital, Yangzhou, Jiangsu Province, China.

**Keywords:** gastric cancer, LncRNA, STARD4-AS1, biomarkers, prognostic gene signature

## Abstract

**INTRODUCTION:**

Gastric cancer (GC) is a lethal malignant tumor necessitating high-sensitivity detection to improve diagnostic accuracy and the prognosis of patients. Alterations in long noncoding RNAs can influence cancer progression through various mechanisms. Our study tried to explore the potential of STARD4 antisense RNA 1 (STARD4-AS1) as a GC biomarker and its mechanism of action in GC development.

**METHODS:**

Pan-cancer analysis using The Cancer Genome Atlas database identified STARD4-AS1. Serum STARD4-AS1 levels in patients with GC were measured by quantitative real-time PCR, and diagnostic efficiency was assessed using receiver operating characteristic curves. Functional inactivation experiments and western blotting evaluated the biological role of STARD4-AS1 in GC cells. Bioinformatics analysis explored its potential role in GC immunotherapy and underlying mechanisms.

**RESULTS:**

Pan-cancer analysis revealed lower overall survival in GC patients with higher STARD4-AS1 expression. Quantitative real-time PCR confirmed the reproducibility and stability of STARD4-AS1 as a marker. Serum STARD4-AS1 levels in patients with GC were significantly higher than those in healthy subjects and gastritis patients. Receiver operating characteristic analysis demonstrated that STARD4-AS1 outperformed carcinoembryonic antigen, carbohydrate antigen 199 , and carbohydrate antigen 724 in differentiating GC from gastritis, with optimal diagnostic power when combined with these markers. Knockdown of STARD4-AS1 inhibited GC cell proliferation and metastasis and inhibited the epithelial-mesenchymal transition process. Biosignature prediction indicated that higher STARD4-AS1 expression could evaluate prognosis, as well as regulate GC progression through phosphatidylinositol-mediated signaling, and transmembrane receptor protein tyrosine phosphatase signaling pathway.

**DISCUSSION:**

Serum STARD4-AS1 may serve as a diagnostic biomarker and oncogene function for GC for improving diagnosis, monitoring progression, and evaluating prognosis of GC.

## INTRODUCTION

Globally, gastric cancer (GC) ranks fifth in incidence and is the third leading cause of cancer-related deaths (1). GC etiology is complex, involving risk factors such as age, genetics, *Helicobacter pylori* infection, and poor dietary habits (2–4). Most cases are diagnosed at advanced stages (5,6), leading to poor treatment outcomes and low overall survival (OS) rates (7). Early diagnostic methods and treatment modalities are insufficient to improve GC prognosis (8,9). Therefore, identifying more sensitive diagnostic biomarkers and conducting in-depth studies on GC development mechanisms are essential.

The discovery of noncoding RNAs (ncRNAs), crucial for regulating cellular activity, has provided new insights into tumor development and therapy. ncRNAs can be classified by size into small ncRNAs (such as microRNAs,  tRNA-derived small RNAs, and piwi-interacting RNAs) and long noncoding RNAs (lncRNAs) (over 200 nucleotides, including pseudogenes and circular RNAs) (10,11). LncRNAs exert their functions through various mechanisms, such as binding to protein complexes (12,13), transcription factors (14,15), RNA-binding proteins (16–18), and nucleic acids (19,20). They can also act as competitive endogenous RNAs or “sponges” for microRNAs (21,22) and directly interact with DNA (23,24). Many lncRNAs are overexpressed in cancer and function as pro-oncogenes (20,25). In addition, lncRNAs have been detected in the body fluids of patients with cancer, suggesting potential use as liquid biopsy markers (26–28). Numerous studies highlight the role of lncRNAs in GC. Zhang et al (29) found that colon cancer-associated transcript-1 in serum and plasma exosomes promotes GC progression through the polypyrimidine tract binding protein 1/pyruvate kinase M2/glycolytic pathway. Wu et al (30) showed that upregulation of small nucleolar host gene 11, induced by transcription factor 7 like 2 transcription, exacerbates oncogenic autophagy and regulates GC malignancy. Fang et al (31) revealed that AlkB homolog 5 downregulates TP53TG1 through m6A modification, which interacts with cancerous inhibitor of protein phosphatase 2A to inhibit the phosphoinositide 3-kinase/protein kinase B pathway and suppress GC development.

Abnormal expression of STARD4 antisense RNA 1 (STARD4-AS1) affects cancer development and progression. Li et al (32) showed that STARD4-AS1, highly expressed in oral squamous cell carcinoma (OSCC), inhibited erastin-induced ferroptosis and influenced cellular phenotypes, suggesting its potential as an OSCC biomarker. In head and neck squamous cell carcinoma (HNSCC), overexpressed STARD4-AS1 correlates with better survival (33). However, the diagnostic potential, mechanism, and prognostic significance of STARD4-AS1 in GC remain unclear.

We identified STARD4-AS1 as significantly overexpressed in GC using pan-cancer analysis from The Cancer Genome Atlas (TCGA) database. We analyzed its diagnostic value as a serum biomarker and explored its role in gastric carcinogenesis and progression. By analyzing serum samples from 129 patients with GC, 55 gastritis patients, and 116 healthy subjects, we established a diagnostic model combining STARD4-AS1 with serum CEA (arcinoembryonic antigen), CA199 (carbohydrate antigen 199), and CA724 (carbohydrate antigen 724) to assess its effectiveness in diagnosing GC. *In vitro* experiments examined the biological role of STARD4-AS1 in GC progression. Bioinformatics analyses were conducted to evaluate its immunotherapeutic potential and mechanisms in GC. The aim of our findings was to enhance understanding of STARD4-AS1 in GC and contribute to further research on metastatic mechanisms and therapeutic prognosis.

## METHODS

### TCGA data analysis

Differential expression data, clinical information (including survival status and OS time), and sample mutation information for STARD4-AS1 were obtained from the TCGA database (https://portal.gdc.cancer.gov/). All analyses were conducted using R software (https://www.r-project.org/).

### Clinical serum samples

Serum specimens from 129 patients with GC (aged 40–84 years, 93 male patients and 36 female patients), 55 patients with gastritis (aged 36–74 years, 36 male patients and 19 female patients), and 116 healthy subjects (aged 23–71 years, 68 male patients and 48 female patients) were provided by the clinical laboratory of Nantong University Hospital between 2018 and 2022. Owing to sample acquisition limitations, the age and sex distribution of the gastritis and healthy subject groups was as close as possible to that of the GC group to minimize potential confounding effects. Among the patients with GC, 53 underwent surgery without previous radiotherapy, chemotherapy, targeted therapy, or immunotherapy. All operated patients underwent open or laparoscopic distal gastrectomy with D1/D2 lymphadenectomy as clinically indicated. The study protocol was reviewed and approved by the Ethics Committee of the Nantong University Affiliated Hospital (Ethics Review No. 2018-L055), and all study investigations followed the provisions of the 1975 Declaration of Helsinki.

### Cell culture

Human GC cell lines (HGC-27, SGC-7901, adenocarcinoma gastric cells [AGS], MKN-1[derived from a poorly differentiated gastric adenocarcinoma], and Beijing Gastric Carcinoma-823 [BGC-823]) and normal human gastric epithelial cells (GES-1) were purchased from the Shanghai Institute of Biological Sciences, Chinese Academy of Sciences (Shanghai, China). Cells were cultured in Roswell Park Memorial Institute 1640 medium with 10% fetal bovine serum (both from Procell Lite Science & Technology Co., Ltd., Wuhan, China) and 1% penicillin-streptomycin (InvivoGen, San Diego, CA, USA). All cell lines were maintained at 37 °C in a 5% CO2 incubator to ensure sterility.

### Total RNA extraction, cDNA synthesis, and quantitative real-time PCR

Total RNA was extracted from serum using the Rapid Blood Total RNA Extraction Kit (BioTeke, Wuxi, Jiangsu, China) and from cells using TRIzol reagent (Invitrogen, Carlsbad, CA, USA). RNA concentrations were approximately 15 ng/μL for serum and 1,500 ng/μL for cellular RNA. Total RNA was reverse transcribed into cDNA (about 500 ng/μL) using the RevertAid RT Reverse Transcription Kit (Thermo Fisher Scientific, Waltham, MA, USA). Quantitative real-time PCR (qRT–PCR) was performed on an Applied Biosystems QuantStudio 5 with a reaction mixture containing 10 μL of 2× ChamQ Universal SYBR Green I quantitative PCR Master Mix (Vazyme Biotech, Nanjing, Jiangsu, China), 0.5 μL of each primer (10 μM), 4 μL of nuclease-free water, and 5 μL of diluted cDNA (500 ng/μL). 18S rRNA served as the internal reference (10 μM). STARD4-AS1 expression was normalized using the 2^−ΔΔCT^ method. Primers were synthesized by RiboBio (Guangzhou, China), and their sequences are listed in Supplementary Table 1, see Supplementary Digital Content 1, http://links.lww.com/CTG/B380.

### Agarose gel electrophoresis

A 2% agarose gel was used to separate DNA samples. Each sample (1 μL) was mixed with 5 μL of 6× DNA loading buffer (Beyotime Biotechnology, Shanghai, China). DNA Ladder (500 bp Plus Marker, Cat. No. C500233, Sangon Biotech) included bands at 50, 100, 150, 200, 250, 300, 400, and 500 bp, with the 250 bp band serving as a reference for yield. DNA fragments were visualized using a ChemiDoc MP gel imaging system (Bio-Rad Laboratories, Hercules, CA, USA).

### Cell transfection

The STARD4-AS1-interfering plasmids (see Supplementary Table 2, Supplementary Digital Content 2, http://links.lww.com/CTG/B381 which demonstrates shRNA sequences.) were obtained from GenePharma (Shanghai, China). When cell density reached approximately 70%, each well of a 6-well plate was transfected with 2,500 ng of sh-NC, sh-1, or sh-2 using 3.75 μL of Lipofectamine 3,000 in 2 mL of complete medium. After 12 hours, the medium was replaced with 2 mL of fresh complete medium containing serum and antibiotics. Cells were collected 48 hours post-transfection for subsequent experiments. Changes in STARD4-AS1 expression levels were detected using qRT-PCR.

### Cell proliferation and colony formation assays

Cell proliferation was assessed by Cell Counting Kit-8 (CCK-8) and colony formation assays. For CCK-8 experiments, cells were seeded in 96-well plates at 3 × 10^3^ cells per well, and CCK-8 assay solution (purchased from Biosharp, Hefei, China) was added at 0, 24, 48, 72, and 96 hours after cell attachment. The plates were incubated for 2 hours under light-protected conditions, and absorbance was measured at 450 and 630 nm using a microplate reader (Thermo Fisher MK3, Waltham, MA, USA). For colony formation assays, 1 × 10^3^ cells per well were seeded in 6-well plates and incubated for 14 days with media changes every 4 days. Colonies were fixed with 4% paraformaldehyde and stained with crystal violet (Beyotime Biotechnology, Shanghai, China). Photographs were taken, and colonies were quantified using ImageJ software (v1.8.0; NIH, Bethesda, MD, USA).

### Transwell migration and invasion analysis

Cell suspensions 48 hours post-transfection were seeded into Transwell upper chambers (8 μm pore size; Corning Life Science, NY, USA) with or without Matrigel (Corning, NY, USA) to assess GC cells' migratory and invasive abilities. For migration assays, 5 × 10^4^ cells per well were added to the upper chambers in serum-free medium, while for invasion assays, 8 × 10^4^ cells per well were used. The lower chambers were filled with medium containing 20% fetal bovine serum. After 24–36 hours of incubation, the Transwell chambers were removed, and the cells were fixed with 4% paraformaldehyde for 15 minutes, followed by staining with 0.2% crystal violet for 15 minutes. Nonmigrated or noninvaded cells were removed from the upper surface using cotton swabs. The chambers were then washed, and the cells were observed, photographed, and counted using an inverted microscope and ImageJ software (v1.8.0; NIH, Bethesda, MD, USA).

### Western blot

Cells were lysed using radioimmunoprecipitation assay buffer (Epizyme, Shanghai, China) supplemented with phenylmethylsulfonyl fluoride (SolarBio, Beijing, China), and total protein concentration was determined by bicinchoninic acid assay. Equal amounts of protein were separated on 10% sodium dodecyl sulfate-polyacrylamide gel electrophoresis gels (Epizyme, Shanghai, China) and transferred to polyvinylidene difluoride membranes (Millipore, MA, USA). Membranes were blocked with 5% nonfat milk and incubated overnight at 4 °C with primary antibodies, including E-cadherin (E-cad), N-cadherin (N-cad), Vimentin (Cell Signaling Technology, MA, USA), and glyceraldehyde-3-phosphate dehydrogenase (GAPDH, Proteintech, Wuhan, China) as an internal control. After washing, membranes were incubated with corresponding secondary antibodies (Cell Signaling Technology, Danvers, MA, USA) for 2 hours at room temperature. Protein bands were visualized using an enhanced chemiluminescence detection kit (Fdbio, Hangzhou, China).

### Bioinformatics analysis

VarScan-processed Mutation Annotation Format data for TCGA-STAD were downloaded from the TCGA database and analyzed using the Maftools R package to identify key gene mutations and plot single nucleotide variant (SNV) distributions (34). Pearson correlation analysis evaluated the relationship between STARD4-AS1 expression and tumor mutational burden (TMB) and microsatellite instability (MSI). The results were visualized using radargrams generated with the “fmsb” R package. The MEM (https://biit.cs.ut.ee/mem/, accessed June 20, 2024) and RIsearch (http://rtools.cbrc.jp/LncRRIsearch, accessed June 20, 2024) databases, commonly used target gene prediction databases for lncRNAs, were used for cross-analysis of STARD4-AS1 target genes (35,36). Gene Oncology (GO) functional and Kyoto Encyclopedia of Genes and Genomes (KEGG) pathway analyses were performed using an online data analysis and visualization platform at https://www.bioinformatics.com.cn (accessed June 20, 2024).

### Statistical analysis

All statistical analyses were performed using SPSS 20.0 (IBM SPSS Statistics, Chicago, IL) and GraphPad Prism V.8.00 (GraphPad Software, La Jolla, CA). Data are presented as the mean ± SD from 3 independent experiments. The Student t-test was used to compare differences between groups. The correlation between STARD4-AS1 expression and clinicopathological parameters was analyzed using the χ^2^ test. The Youden index was used to determine the optimal threshold for STARD4-AS1. The thresholds for CEA, CA199, and CA724 were set at 5 ng/mL, 37 U/mL, and 10 U/mL, respectively, based on the reference ranges from Nantong University Hospital. The diagnostic efficacy of STARD4-AS1 in GC serum was evaluated by constructing a receiver operating characteristic (ROC) curve and calculating the area under the curve (AUC). Statistical significance threshold was set at *P* < 0.05 (ns, *P* > 0.05,*P < 0.05, **P < 0.01, ***P < 0.001, ****P < 0.0001).

## RESULTS

### TCGA database analysis STARD4-AS1 is highly expressed in GC and is associated with poorer prognosis

**Figure 1.**
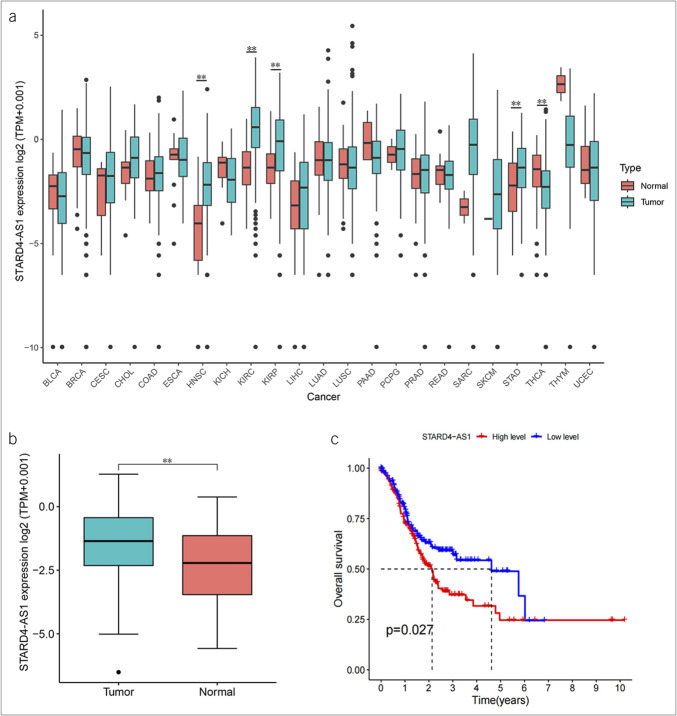
STARD4-AS1 expression was upregulated in gastric cancer (GC) and associated with poorer prognosis. (**a**) Boxplot of the expression level of STARD4-AS1 in cancers and normal tissues of 33 cancers from The Cancer Genome Atlas (TCGA) database. (**b**) The expression of STARD4-AS1 in GC tissues using data from TCGA. (**c**) Kaplan-Meier curves for correlation analyses of STARD4-AS1 expression and GC patients' overall survival in the TCGA database. BLCA, bladder urothelial carcinoma; BRCA, breast invasive carcinoma; CESC, cervical squamous cell carcinoma; CHOL, cholangiocarcinoma; COAD, colon adenocarcinoma; ESCA, esophageal carcinoma; HNSC, head and neck squamous cell carcinoma, KICH, kidney chromophobe; KIRC, kidney renal clear-cell carcinoma; KIRP, kidney renal papillary cell carcinoma; LIHC, liver hepatocellular carcinoma; LUAD, lung adenocarcinoma; LUSC, lung squamous cell carcinoma; PAAD, pancreatic adenocarcinoma; PCPG, pheochromocytoma and paraganglioma; PRAD, prostate adenocarcinoma; READ, rectum adenocarcinoma; SARC, sarcoma; SKCM, skin cutaneous melanoma; STAD, stomach adenocarcinoma; THCA, thyroid carcinoma; THYM, thymoma; UCEC, uterine corpus endometrial carcinoma.

We analyzed STARD4-AS1 expression across various cancers using the TCGA database. STARD4-AS1 was significantly upregulated in HNSC (head and neck squamous cell carcinoma), KIRC (kidney renal clear-cell carcinoma), KIRP (kidney renal papillary cell carcinoma), and STAD (stomach adenocarcinoma), whereas downregulated in thyroid carcinoma (Figure 1a). In GC, STARD4-AS1 levels were markedly higher in tumor tissues compared with normal tissues (Figure 1b). Kaplan-Meier survival analysis from the TCGA database revealed that high STARD4-AS1 expression correlated with poorer OS (*P* = 0.027) (Figure 1c). Therefore, high expression of STARD4-AS1 in GC is associated with an unfavorable prognosis.

### Comprehensive evaluation of STARD4-AS1 as an assay for biomarkers

We created 10-fold, 10^2^-fold, 10^3^-fold 10^4^-fold, and 10^5^-fold dilutions of STARD4-AS1 cDNA and performed qRT-PCR. The results demonstrated a good linearity, indicating that the method is suitable for laboratory analysis (see Supplementary Figure 1, Supplementary Digital Content 3, A, B, http://links.lww.com/CTG/B382). Agarose gel electrophoresis of the qRT-PCR products revealed a single clear band at approximately 193 bp, confirming accuracy (see Supplementary Figure 1, Supplementary Digital Content 3, C, http://links.lww.com/CTG/B382). Sanger sequencing of the qRT-PCR product revealed a 193 bp sequence identical to STARD4-AS1, further validating the accuracy of the qRT-PCR method (see Supplementary Figure 1, Supplementary Digital Content 3, D, http://links.lww.com/CTG/B382).

### Expression levels of STARD4-AS1 in GC sera and their correlation with clinicopathologic parameters

**Figure 2.**
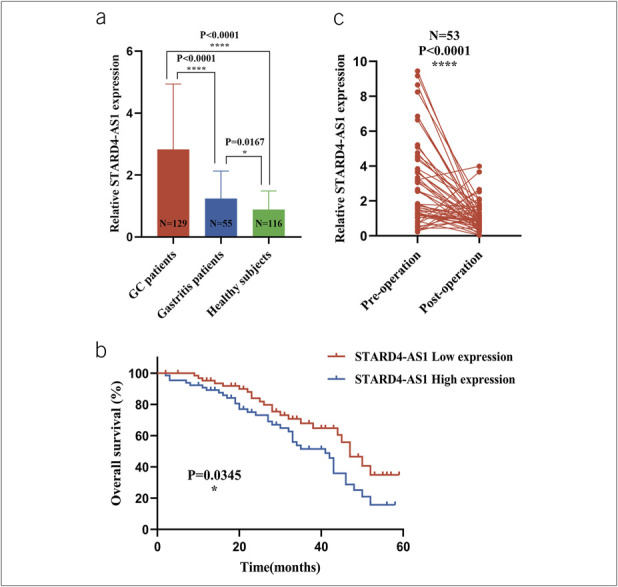
The prognostic and diagnostic potential of serum STARD4-AS1. (**a**) Detection of serum expression of STARD4-AS1 in patients with GC (n = 129), gastritis patients (n = 55), and healthy subjects (n = 116). (**b**) Altered expression of serum STARD4-AS1 in 53 paired samples preoperatively and postoperatively. (**c**) The survival curve of patients with GC. *P < 0.05, ****P < 0.001, NS means no statistically difference. GC, gastric cancer.

qRT-PCR analysis of clinical serum specimens showed that STARD4-AS1 levels were significantly elevated in patients with GC (*P* < 0.0001) and slightly higher in gastritis patients (*P* = 0.0167) compared with healthy subjects (Figure 2a). χ^2^ analysis revealed that the high expression level of serum STARD4-AS1 expression correlated with T stage, lymph node metastasis, and tumor-node-metastasis (TNM) stage but not with sex, age, tumor size, differentiation degree, neuro/vascular invasion, Lauren staging, or other clinicopathologic parameters (Table 1). Kaplan-Meier analysis indicated that patients with high STARD4-AS1 expression (≥2.354, n = 64) had a lower survival rate than those with low expression (<2.354, n = 65) according to the median serum expression of STARD4-AS1(Figure 2b). In addition, in 53 patients with GC who underwent tumor resection, postoperative STARD4-AS1 levels decreased significantly, returning to levels similar to healthy subjects (*P* < 0.0001) (Figure 2c). These findings suggest that serum STARD4-AS1 can serve as a marker for GC diagnosis and tumor dynamics monitoring.

**Table 1.** The Correlation between STARD4-AS1 expression and clinicopathologic parameters of patients with gastric cancer

Parameter	No. of patients	STARD4-AS1 (high)	STARD4-AS1 (low)	*P* value
Sex				
Male	93	47	46	0.956
Female	36	18	18	
Age (yr)				
<60	35	17	18	0.801
≥60	94	48	46	
Tumor size (cm)				
<5	79	43	36	0.248
≥5	50	22	28	
Differentiation grade				
Well-moderate	55	28	27	0.919
Poor-undifferentiation	74	37	37	
T stage				
T1-T2	41	28	13	0.005**
T3-T4	88	37	51	
Lymph node status				
Positive	89	52	37	0.006**
Negative	40	52	20	
TNM stage				
Ⅰ–Ⅱ	74	44	30	0.009**
Ⅲ–Ⅳ	55	35	20	
Nerve/vascular invasion				
Positive	75	38	37	0.940
Negative	54	27	27	
Intestinal type	54	26	28	
Lauren classification				
Mixed type	39	21	18	0.862
Diffuse type	36	18	18	

Statistical analyses were conducted using the Pearson χ^2^ test.

**P < 0.01.

### Potential diagnostic value of serum STARD4-AS1 for GC

**Figure 3.**
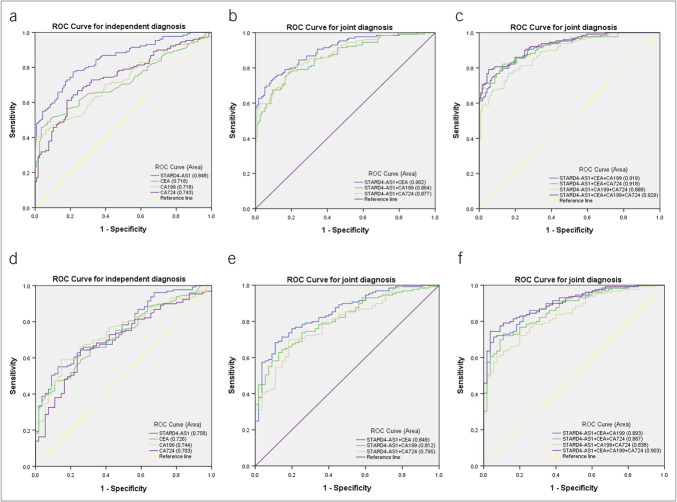
Evaluation of the diagnostic efficacy of serum STARD4-AS1 in GC. (**a**) ROC analysis of STARD4-AS1, CEA, CA199, and CA724 in the independent diagnosis of patients with GC and healthy subjects. (**b, c**) ROC analysis of STARD4-AS1, CEA, CA199, and CA724 in the joint diagnosis of patients with GC and healthy subjects. (**d**) ROC analysis of STARD4-AS1, CEA, CA199, and CA724 in the independent diagnosis of patients with GC and gastritis patients. (**e, f**) ROC analysis of STARD4-AS1, CEA, CA199, and CA724 in the joint diagnosis of patients with GC and gastritis patients. GC, gastric cancer; ROC, receiver operating characteristic.

ROC curve analysis of STARD4-AS1 in the serum of 129 patients with GC demonstrated an AUC of 0.848 (95% confidence interval [CI]: 0.801–0.895), surpassing traditional markers CEA (AUC: 0.718, 95% CI, 0.654–0.783), CA199 (AUC: 0.718, 95% CI, 0.654–0.781), and CA724 (AUC: 0.743, 95% CI, 0.682–0.805) (Figure 3a). At a cutoff value of 1.224 (Youden index: 0.559), STARD4-AS1 achieved 78% sensitivity (SEN) and specificity (SPE), with overall accuracy (ACCU) at 78%, positive predictive value at 80%, and negative predictive value at 76% (Table 2). Combining STARD4-AS1 with CEA, CA199, and CA724 further improved diagnostic ACCU, achieving the highest AUC of 0.929 when all 4 markers were used together (Figure 3b,c; Table 2).

**Table 2.** Combination of serum STARD4-AS1, CEA, CA199, and CA724 levels significantly improves the diagnostic sensitivity between patients with GC and healthy subjects

	AUC (95% CI)	SEN	SPE	ACCU	PPV	NPV
STARD4-AS1	0.848 (0.801–0.895)	0.78 (100/129)	0.78 (91/116)	0.78 (191/245)	0.80 (100/125)	0.76 (91/120)
CEA	0.718 (0.654-0.783)	0.52 (67/129)	0.90 (104/116)	0.70 (171/245)	0.85 (67/79)	0.63 (104/166)
CA199	0.718 (0.654-0.718)	0.46 (59/129)	0.91 (105/116)	0.67 (164/245)	0.84 (59/70)	0.60 (105/175)
CA724	0.743 (0.682-0.805)	0.59 (76/129)	0.82 (95/116)	0.70 (171/245)	0.78 (76/97)	0.64 (95/148)
STARD4-AS1+CEA	0.902 (0.865-0.939)	0.91 (117/129)	0.72 (83/116)	0.82 (202/245)	0.78 (117/150)	0.87 (83/95)
STARD4-AS1+CA199	0.864 (0.820-0.908)	0.84 (108/129)	0.75 (87/116)	0.80 (195/245)	0.79 (108/137)	0.81 (87/108)
STARD4-AS1+CA724	0.877 (0.836-0.919)	0.90 (116/129)	0.66 (76/116)	0.78 (192/245)	0.74 (116/156)	0.85 (76/89)
STARD4-AS1+CEA+CA199	0.919 (0.886-0.951)	0.95 (123/129)	0.68 (79/116)	0.82 (202/245)	0.77 (123/160)	0.93 (79/85)
STARD4-AS1+CEA+CA724	0.918 (0.885-0.951)	0.95 (123/129)	0.59 (68/116)	0.78 (191/245)	0.72 (123/171)	0.92 (68/74)
STARD4-AS1+CA199+CA724	0.888 (0.849-0.927)	0.91 (118/129)	0.62 (72/116)	0.78 (190/245)	0.73 (118/162)	0.87 (72/83)
STARD4-AS1+CEA+CA199+CA724	0.929 (0.900-0.959)	0.97 (125/129)	0.55 (64/116)	0.77 (189/245)	0.71 (125/177)	0.94 (64/68)

SEN, sensitivity; SPE, specificity; ACCU, overall accuracy; PPV, positive predictive value; NPV, negative predictive value.

In addition, ROC analysis comparing 129 patients with GC with 55 gastritis patients showed that STARD4-AS1 had an AUC of 0.758 (95% CI, 0.687–0.829), was higher than that of CEA (0.726, 95% CI: 0.652–0.800), CA199 (0.744, 95% CI: 0.671–0.816), and CA724 (0.703, 95% CI: 0.625–0.782) (Figure 3d). At a cutoff value of 2.090 (Youden index: 0.423), STARD4-AS1 achieved 55% SEN and 87% SPE (Table 3), demonstrating its potential to differentiate between patients with GC and gastritis. Diagnostic efficacy increased when combining STARD4-AS1 with other markers, reaching an AUC of 0.903 (95% CI: 0.860–0.945) with 93% SEN when all 4 markers were used together (Figure 3e,f; Table 3).

**Table 3.** Combination of serum STARD4-AS1, CEA, CA199, and CA724 levels significantly improves the diagnostic sensitivity between patients with GC and gastritis patients

	AUC (95% CI)	SEN	SPE	ACCU	PPV	NPV
STARD4-AS1	0.758 (0.687-0.829)	0.55 (71/129)	0.87 (48/55)	0.65 (119/184)	0.91 (71/78)	0.45 (48/106)
CEA	0.726 (0.652-0.800)	0.52 (67/129)	0.80 (44/55)	0.60 (111/184)	0.86 (67/78)	0.42 (44/106)
CA199	0.744 (0.671-0.816)	0.46 (59/129)	0.89 (49/55)	0.59 (108/184)	0.91 (59/65)	0.41 (49/119)
CA724	0.703 (0.625-0.782)	0.59 (76/129)	0.76 (42/55)	0.64 (118/184)	0.85 (76/89)	0.44 (42/95)
STARD4-AS1+CEA	0.849 (0.793-0.905)	0.81 (104/129)	0.71 (39/55)	0.78 (143/184)	0.87 (104/120)	0.61 (39/64)
STARD4-AS1+CA199	0.812 (0.750-0.873)	0.68 (88/129)	0.76 (42/55)	0.71 (130/184)	0.87 (88/101)	0.51 (42/83)
STARD4-AS1+CA724	0.795 (0.729-0.861)	0.80 (103/129)	0.67 (37/55)	0.76 (140/184)	0.85 (103/121)	0.59 (37/63)
STARD4-AS1+CEA+CA199	0.893 (0.848-0.939)	0.91 (117/129)	0.62 (34/55)	0.82 (151/184)	0.85 (117/138)	0.74 (34/46)
STARD4-AS1+CEA+CA724	0.867 (0.815-0.919)	0.91 (117/129)	0.56 (31/55)	0.80 (148/184)	0.83 (117/141)	0.72 (31/43)
STARD4-AS1+CA199+CA724	0.838 (0.782-0.895)	0.55 (71/129)	0.87 (48/55)	0.65 (119/184)	0.91 (71/78)	0.45 (48/106)
STARD4-AS1+CEA+CA199+CA724	0.903 (0.860-0.945)	0.52 (67/129)	0.80 (44/55)	0.60 (111/184)	0.86 (67/78)	0.42 (44/106)

SEN, sensitivity; SPE, specificity; ACCU, overall accuracy; PPV, positive predictive value; NPV, negative predictive value.

We concluded that STARD4-AS1 has diagnostic potential for GC. Although STARD4-AS1 shows moderate ACCU in distinguishing GC from gastritis, its clinical value is enhanced when combined with conventional biomarkers. It may also be useful in predicting malignant progression and the prognostic status of GC.

### STARD4-AS1 promotes GC cell proliferation, migration, and invasion

**Figure 4.**
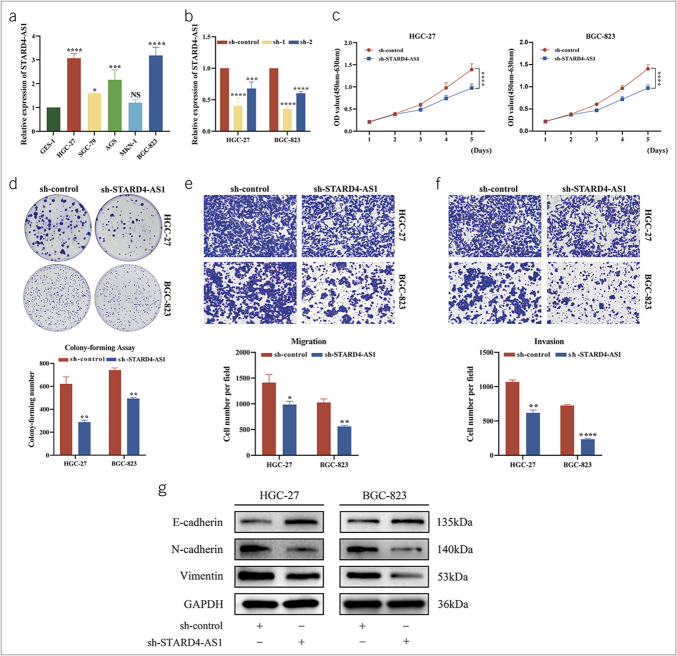
STARD4-AS1 exerts oncogenic roles in GC *in vitro*. (**a**) The expression level of STARD4-AS1 in GC cells was detected by qRT-PCR. (**b**) GC cell lines HGC-27 and BGC-823 cells were transfected with STARD4-AS1 shRNA or control shRNA, and the knockdown efficiency was detected by qRT-PCR. (**c, d**) The effects of transfection with STARD4-AS1 shRNA1 or control shRNA on the proliferation of HGC-27 and BGC-823 were evaluated by CCK-8 and cell colony formation assay. (**e, f**) The metastasis ability of HGC-27 and BGC-823 cells after STARD4-AS1 knockdown was measured by using Transwell assay. (**g**) After the downregulation of STARD4-AS1, major epithelial-mesenchymal transition (EMT) proteins were expressed in HGC-27 and BGC-823 cells. Data were expressed as mean ± SD, n = 3. *P < 0.05, **P < 0.01, ***P < 0.001, ****P < 0.001. GC, gastric cancer; HGC, human GC cell lines; qRT, quantitative real-time.

We determined the expression levels of STARD4-AS1 in 5 GC cell lines (HGC-27, SGC-7901, AGS, MKN-1, and BGC-823) and the gastric epithelial cell line GES-1 using qRT-PCR. STARD4-AS1 was significantly upregulated in HGC-27 and BGC-823 compared with GES-1 (Figure 4a). We designed 2 shRNAs to knock down STARD4-AS1 in HGC-27 and BGC-823 cells, with only sh-1 (STARD4-AS1-Homo-3506) effectively reducing its expression (Figure 4b). Therefore, we selected the HGC-27 and BGC-823 cell lines and sh-1 for further knockdown experiments.

CCK-8 and colony-forming assays showed that knocking down STARD4-AS1 significantly reduced the proliferative activity of both cell lines (Figure 4c,d). Transwell assays further elucidated that the knockdown of STARD4-AS1 significantly decreased the migration and invasion of GC cells (Figure 4e,f). We detected the expression levels of E-cad, N-cad, and Vimentin protein in GC cells of HGC-27 and BGC-823 with STARD4-AS1 knocked down by western blot. Compared with the sh-control group, we found that after knockdown of STARD4-AS1, the E-cad content in HGC-27 and BGC-823 cells increased, whereas the expression levels of N-cad and Vimentin decreased (Figure 4g). These data suggest that STARD4-AS1 promotes GC cell proliferation, migration, and invasion, consistent with its expression patterns observed in the TCGA database and serum samples.

### SNV analysis of STAD patients and association between STARD4-AS1 and TMB

Missense mutations are the most common type of SNVs in STAD patients, with C>T transitions being the predominant single nucleotide polymorphisms type (see Supplementary Figure 2, Supplementary Digital Content 4, A, http://links.lww.com/CTG/B383). On average, each sample contained 97 variants, and the top 5 mutated genes were Titin (TTN) (51%), Mucin 16 (MUC16) (31%), TP53 (46%), low-density lipoprotein receptor-related protein 1B (LRP1B) (27%), and AT-rich interaction domain 1A (ARID1A) (27%). Of the 431 STAD samples analyzed, 397 (92.11%) harbored mutations (see Supplementary Figure 2, Supplementary Digital Content 4, B, http://links.lww.com/CTG/B383). The transition plot showed that substitutions were more frequent than reversals (see Supplementary Figure 2, Supplementary Digital Content 4, C, http://links.lww.com/CTG/B383). A high proportion of mutated genes synergize in the top 10 mutation rates, with a bias toward pairs of genes that also have mutations (see Supplementary Figure 2, Supplementary Digital Content 4, D, http://links.lww.com/CTG/B383). TMB analysis is crucial for predicting tumor immunotherapy efficacy. STARD4-AS1 expression was significantly associated with TMB and MSI in the TCGA cohort (see Supplementary Figure 2, Supplementary Digital Content 4, E, F, http://links.lww.com/CTG/B383).

### Downstream forecast for STARD4-AS1

**Figure 5.**
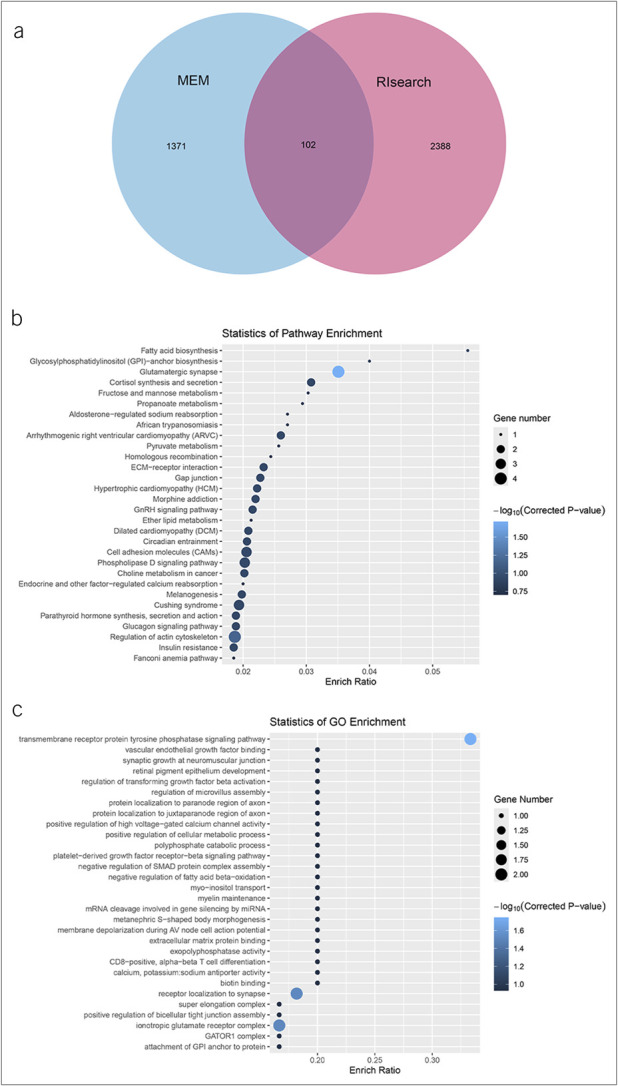
Prediction of STARD4-AS1 target gene and target gene KEGG and GO enrichment analysis. (**a**) Prediction of target genes for STARD4-AS1 based on the MEM and RIsearch databases. (**b**) KEGG biological pathway functional enrichment analysis of the target genes of STARD4-AS1. (**c**) GO functional enrichment analysis of the target genes of STARD4-AS1. GO, Gene Oncology; KEGG, Kyoto Encyclopedia of Genes and Genomes.

102 target genes at the intersection section of the 2 databases (MEM and RIsearch) are likely regulated by STARD4-AS1 (Figure 5a). KEGG pathway enrichment analysis revealed significant involvement in herpes simplex virus 1 infection and glutamatergic synapse signaling pathway (Figure 5b). In addition, GO functional enrichment analysis indicated that these potential target genes may play roles in phosphatidylinositol-mediated signaling and transmembrane receptor protein tyrosine phosphatase signaling pathway, among others (Figure 5c). Although these analyses provide valuable insights, further investigation is needed to elucidate the underlying regulatory mechanisms of STARD4-AS1.

## DISCUSSION

GC, characterized by its high mortality rate, imposes significant health and economic burdens globally (37). This malignancy typically evolves through a gastritis-atrophy-metaplasia-dysplasia sequence, with chronic inflammation serving as critical pathogenic groundwork (38). GC progression involves the accumulation of multigene mutations and complex epigenetic changes (39). Current diagnostic methods have a relatively low detection rate for early-stage GC, particularly in high-risk populations with premalignant conditions such as gastritis, leading to many cases being diagnosed at advanced stages with poorer survival outcomes (7,40). Therefore, the development of liquid biopsy markers for GC and the study of its pathogenesis has garnered considerable attention.

LncRNAs, as a class of ncRNAs, have become one of the most active areas of cancer research (41). They are involved in various stages of cancer development (42), and recent studies show their close association with cancer progression (40,43), potential as biomarkers (44,45). Although STARD4-AS1 has been identified as an oncogene promoter in OSCC and head and neck squamous cell carcinoma (32,33), its role as a diagnostic marker in GC remains limited. Based on TCGA database analysis, we found that STARD4-AS1 is significantly upregulated in GC tissues, and high expression correlates with poorer OS. We therefore aimed to evaluate the diagnostic potential of serum STARD4-AS1 in GC, comparing it with conventional tumor markers (CEA, CA199, and CA724) (46). Our results showed that serum STARD4-AS1 levels were markedly elevated in patients with GC compared with healthy subjects, with intermediate levels observed in patients with gastritis. ROC curve analysis demonstrated that STARD4-AS1 outperformed CEA, CA199, and CA724 in distinguishing between patients with GC and healthy subjects. Although its ACCU in differentiating GC from gastritis was moderate, this finding aligns with the known biological continuum between these conditions (47). Importantly, combining STARD4-AS1 with traditional markers significantly improved diagnostic performance. Accumulating evidence indicates that lncRNAs play pivotal roles in inflammatory pathways and the epithelial-mesenchymal transition (EMT), both of which are central to the progression from gastritis to GC (48,49). In our study, downregulation of STARD4-AS1 was associated with suppression of EMT, suggesting a functional link between this lncRNA and gastric carcinogenesis. These findings imply that STARD4-AS1 may serve not only as a cancer-associated biomarker but also as an indicator of inflammation-driven gastric pathology. Although further mechanistic studies are warranted to elucidate its role in early disease stages, our data highlight the potential of STARD4-AS1 as a supplementary diagnostic tool, particularly for individuals at high risk of developing precancerous lesions. Moreover, its dynamic expression in gastritis and after surgery suggests a role in monitoring disease recurrence and prognosis.

We also found that higher serum STARD4-AS1 expression levels correlated significantly with T-stage, lymph node metastasis, and TNM stage, suggesting its potential role in predicting tumor progression. Previous studies have shown significant correlations between the expression of circulating biomarkers in blood and cells (50), and circulating lncRNAs are known to be present in body fluids in a stable form (51). We examined the expression of STARD4-AS1 in GES-1, HGC-27, SGC-7901, AGS, MKN-1, and BGC-823 cell lines. Relative to GES-1, STARD4-AS1 was overexpressed in tumor cell lines, particularly associated with metastasis. To test our hypothesis that STARD4-AS1 originates from tumor cell secretion and promotes metastasis, we performed functional inactivation experiments in HGC-27 and BGC-823 cells. Knockdown of STARD4-AS1 reduced cell proliferation, migration, and invasion. Furthermore, western blot results showed that downregulation of STARD4-AS1 inhibited the EMT process. Our *in vitro* findings suggest that STARD4-AS1 acts as an oncogene in GC that promotes cancer cell growth, invasion, and metastasis. Studies on antisense lncRNAs indicate their role in promoting cancer metastasis (52,53), supporting the potential significance of STARD4-AS1 in GC development.

Tumor development and progression are closely linked to genomic changes (54). SNV analysis of STAD samples revealed that the top 3 mutated genes were TTN, MUC16, and TP53. These mutations can significantly influence GC treatment and prognosis. Our study explored STARD4-AS1 as an oncogene in GC (55), suggesting its potential as a marker for disease progression and prognosis. TMB has been shown to affect the clinical efficacy of tumor-infiltrating immune cells and immunotherapy (56). High TMB in various tumors indicates better outcomes with immune checkpoint inhibitors compared with chemotherapy (57). In our analysis of STAD cases from the TCGA database, STARD4-AS1 expression was significantly correlated with TMB and MSI. However, the present data still do not directly predict response to immunotherapy; further mechanistic studies and validation in immunotherapy cohorts are needed to assess whether STARD4-AS1 regulates response to therapy.

Moreover, KEGG pathway enrichment analysis and GO functional enrichment analysis preliminarily predicted the downstream targets of STARD4-AS1, suggesting its association with cancer development and progression. STARD4-AS1 was significantly enriched in pathways such as phosphatidylinositol-mediated signaling and transmembrane receptor protein tyrosine phosphatase signaling pathway, which are known to promote cellular differentiation, migration, and proliferation. Dysregulation of these pathways contributes to tumor development (58,59). The involvement of STARD4-AS1 in these pathways underscores its importance as an oncogenic lncRNA in GC and highlights its potential as a diagnostic and prognostic biomarker and therapeutic target for GC. However, further studies are needed to validate the molecular mechanisms of STARD4-AS1 in GC by examining its direct interactions with specific target genes and observing the effects on gene expression and pathway activities. This will provide deeper insights into GC pathogenesis and the relationship between target gene functions and GC pathophysiology.

Our study has several limitations. STARD4-AS1 data were sourced from databases and lacked the credibility of clinically sourced lncRNAs identified through high-throughput sequencing of GC tissue or serum. Although STARD4-AS1 expression was analyzed in serum samples, matched tumor and adjacent normal tissue samples were not available for validation in this study. Future work should include matched tissue and serum samples to confirm the correlation between tissue expression and circulating STARD4-AS1 levels. In addition, STARD4-AS1 diagnostic performance in distinguishing GC from gastritis remains moderate, underscoring the need for further refinement in SPE. Although *H. pylori* is a key driver of gastritis and GC by modulating lncRNA expression (60,61), we lacked data on *H. pylori* infection status in our cohort. The potential influence of *H. pylori* on STARD4-AS1 expression warrants further investigation. Furthermore, a large multicenter study is needed to validate our findings, as current limitations include sample size, incomplete age-matching and sex-matching, regional diversity, and lack of detailed clinical annotations such as tumor anatomical location and proton-pump inhibitor use. Although efforts were made to enroll controls with a comparable age distribution to GC patients, strict matching was not fully achieved because of sample availability. These factors may influence biomarker expression and interpretation and will be prospectively addressed in future investigations. The potential correlation between tumor location and STARD4-AS1 expression will also be explored in our future studies. The mechanisms by which STARD4-AS1 contributes to GC development, as well as its potential implications for treatment response and prognosis, require further investigation. We aim to provide a comprehensive summary and discussion of the role of STARD4-AS1 in GC to facilitate its translational application to the clinic, which is our regret and a direction for future research.

In summary, STARD4-AS1, overexpressed in GC, was identified as a potential marker for GC detection. Our results indicate that elevated STARD4-AS1 levels effectively differentiate patients with GC from those with gastritis and healthy subjects, with higher SEN than CEA, CA199, and CA724. The correlation between STARD4-AS1 and pathological data reflects its predictive value for GC progression. STARD4-AS1 promotes GC cell proliferation and migration, confirming its role as an oncogene. Moreover, STARD4-AS1 may be involved in key mechanisms of GC development, particularly those related to tumor mutations and genomic instability. Targeting STARD4-AS1 could influence GC treatment prognosis. Therefore, STARD4-AS1 is anticipated to serve as a biomarker for diagnosing GC, assessing prognosis, and predicting treatment efficacy.

## CONFLICTS OF INTEREST

**Guarantors of the article:** Shaoqing Ju, PhD.

**Specific author contributions:** All the authors are responsible for ensuring the accuracy and integrity of all aspects of the work and have approved the manuscript for publication. The contributions of the authors are as follows: X.C. and M.C.: wrote the original manuscript, conceptualized the experiment, and performed formal data analysis. X.Q.: revised the manuscript and contributed to the experimental design. X.L. and M.Z.: participated in the investigation. X.S.: revised the manuscript and managed the project. S.J.: revised the manuscript, managed the project, conducted investigations, acquired resources, and secured funding. All authors read and approved the final manuscript.

**Financial support:** This work was supported by the National Natural Science Foundation of China (No. 82272411, 82472356), Jiangsu Provincial Key Medical Discipline (Laboratory) ZDXK202240, Science and Technology Project of Jiangsu Province (BE2023741), the Postgraduate Research & Practice Innovation Program of Jiangsu Province (KYCX24_3586), and Foundation of Jiangsu Province Research Hospital (YJXYY202204-XKB16).

**Potential competing interests:** None to report.

**Study Highlights**

**WHAT IS KNOWN**

✓ LncRNA in body fluids of patients with cancer can be used as diagnostic markers.
✓ The diagnostic potential, mechanism of action, and prognosis significance of STARD4-AS1 in GC remain unclear.
✓ The development and progression of OSCC and NHSCC may be related to the abnormal expression of STARD4-AS1.

**WHAT IS NEW HERE**

✓ STARD4-AS1 is upregulated in GC and associated with lower overall survival and poorer prognosis in patients.
✓ Serum STARD4-AS1 shows higher diagnostic accuracy than traditional markers for GC.
✓ STARD4-AS1 promotes GC cell proliferation, migration, and invasion.
✓ STARD4-AS1 expression is significantly associated with TMB and MSI.

## Supplementary Material

**Figure:**
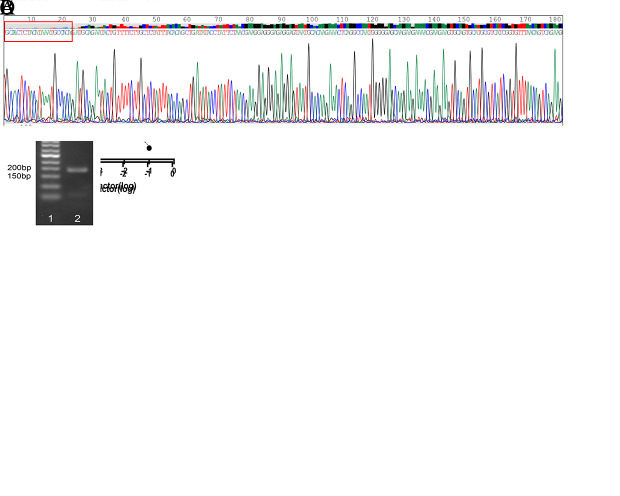


**Figure:**
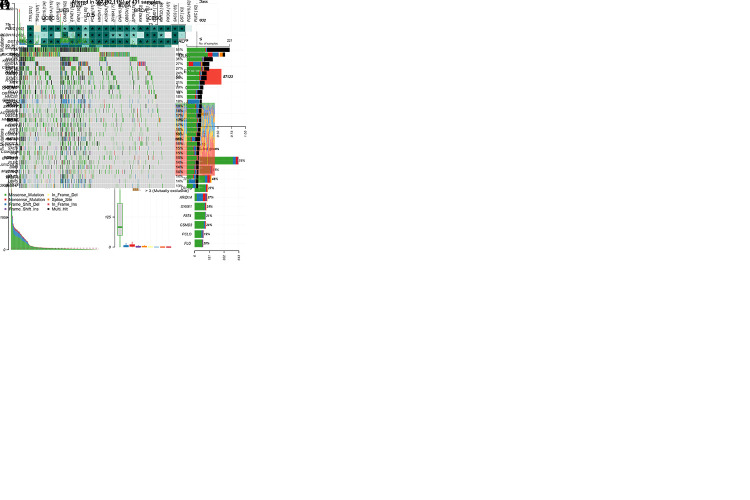


## ABBREVIATIONS:

ACCU: overall accuracy
AGS: adenocarcinoma gastric cells
ARID1A: AT-rich interaction domain 1A
AUC: area under the curve
BGC-823: Beijing Gastric Carcinoma-823
CA199: carbohydrate antigen 199
CA724: carbohydrate antigen 724
CCK-8: Cell Counting Kit-8
CEA: carcinoembryonic antigen
CI: confidence interval
E-cad: E-cadherin
EMT: epithelial-mesenchymal transition
GAPDH: glyceraldehyde-3-phosphate dehydrogenase
GC: gastric cancer
GES-1: gastric epithelial cells
GO: Gene Oncology
HNSC: head and neck squamous cell carcinoma
HNSCC: head and neck squamous cell carcinoma
KEGG: Kyoto Encyclopedia of Genes and Genomes
KIRC: kidney renal clear-cell carcinoma
KIRP: kidney renal papillary cell carcinoma
lncRNAs: long non-coding RNAs
LRP1B: Low-density lipoprotein receptor-related protein 1B
MEM: Multi Experiment Matrix
MSI: microsatellite instability
MUC 16: Mucin 16
N-cad: N-cadherin
ncRNAs: noncoding RNAs
NPV: negative predictive value
OS: overall survival
OSCC: oral squamous cell carcinoma
PPV: positive predictive value
qRT-PCR: quantitative real-time PCR
ROC: receiver operating characteristic
SEN: sensitivity
SNV: single nucleotide variant
SPE: specificity
STAD: stomach adenocarcinoma
TCGA: The Cancer Genome Atlas
TMB: tumor mutational burden
TNM: tumor-node-metastasis
TTN: Titin
