# Supplementary material for: Serum STARD4-AS1 as a Novel Marker for Gastric Cancer Diagnosis and Promotes Gastric Cancer Progression
Source: Clin Transl Gastroenterol. 2025 Sep 3;16(11):e00915. doi: 10.14309/ctg.0000000000000915 (PMC12637328; doi:10.14309/ctg.0000000000000915)
Supplement: Supplementary file 1 [file ct9-16-e00915-s001.docx]

**Supplemental Table 1, Supplemental Digital Content 1.** The primer sequences used for qRT-PCR.

| Name | Sequence |
| --- | --- |
| STARD4-AS1-F | GCAACTCTACATAAATGCCACA |
| STARD4-AS1-R | TGAACTGTTTCGCTTCTGACT |
| 18S rRNA-F | CGCTCGCTCCTCTCCTACTT |
| 18S rRNA-R | CGGGTTGGTTTTGATCTGATAA |
