# Supplementary material for: Serum STARD4-AS1 as a Novel Marker for Gastric Cancer Diagnosis and Promotes Gastric Cancer Progression
Source: Clin Transl Gastroenterol. 2025 Sep 3;16(11):e00915. doi: 10.14309/ctg.0000000000000915 (PMC12637328; doi:10.14309/ctg.0000000000000915)
Supplement: Supplementary file 2 [file ct9-16-e00915-s002.docx]

**Supplemental Table 2, Supplemental Digital Content 2.** shRNA sequence.

| Name | Sequence |
| --- | --- |
| sh1 (STARD4-AS1-Homo-3506) | GCTGCCTTAAGAGAATTATGC |
| sh2 (STARD4-AS1-Homo-664) | GAACCCACCAGCCAAATAAGC |
